# Effects of anandamide in migraine: data from an animal model

**DOI:** 10.1007/s10194-010-0274-4

**Published:** 2011-02-18

**Authors:** Rosaria Greco, Antonina Stefania Mangione, Giorgio Sandrini, Mauro Maccarrone, Giuseppe Nappi, Cristina Tassorelli

**Affiliations:** 1 Headache Science Centre, IRCCS “National Neurological Institute C. Mondino” Foundation, University of Pavia, Via Mondino, 2, 27100 Pavia, Italy; 2 University Centre for the Study of Adaptive Disorder and Headache (UCADH), University of Pavia, Pavia, Italy; 3 University of Rome “La Sapienza”, Rome, Italy; 4 European Center for Brain Research (CERC)/IRCCS S. Lucia Foundation, Rome, Italy; 5 Department of Biomedical Sciences, University of Teramo, Teramo, Italy

**Keywords:** Nitroglycerin, Migraine, AEA, Formalin test

## Abstract

Systemic nitroglycerin (NTG) produces spontaneous-like migraine attacks in migraine sufferers and induces a condition of hyperalgesia in the rat 4 h after its administration. Endocannabinoid system seems to be involved in the modulation of NTG-induced hyperalgesia, and probably, in the pathophysiological mechanisms of migraine. In this study, the analgesic effect of anandamide (AEA) was evaluated by means of the formalin test, performed in baseline conditions and following NTG-induced hyperalgesia in male Sprague–Dawley rats. AEA was administered 30 min before the formalin injection. In addition, the effect of AEA (administered 30 min before NTG injection) was investigated on NTG-induced Fos expression and evaluated 4 h following NTG injection. AEA induced a significant decrease in the nociceptive behavior during both phases of the formalin test in the animals treated with vehicle, while it abolished NTG-induced hyperalgesia during the phase II. Pre-treatment with AEA significantly reduced the NTG-induced neuronal activation in nucleus trigeminalis caudalis, confirming the results obtained in our previous study, and in area postrema, while the same treatment induced an increase of Fos expression in paraventricular and supraoptic nuclei of the hypothalamus, parabrachial nucleus, and periaqueductal grey. The study confirms that a dysfunction of the endocannabinoid system may contribute to the development of migraine attacks and that a pharmacological modulation of CB receptors can be useful for the treatment of migraine pain.

## Introduction

Alterations in the endocannabinoid levels have been found in animal models of pain, neurological and neurodegenerative states, disorders and inflammatory conditions [1, 2]. There is strong evidence that cannabinoids (CB) can induce antinociceptive effects in models of phasic or tonic pain, through activation of CB receptors located on neurons both within and outside the brain and spinal cord [3]. It has been shown that CB suppress spinal Fos expression, a neurochemical marker of neuronal activation [4], in a variety of animal models of persistent pain [5, 6]. The role of the endocannabinoid system in the pathogenesis of headaches has been recently put under scrutiny. Migraine may be caused by cerebral vasodilatation or by abnormal neurological firing or by neurogenic dural inflammation [7]. Trigeminal sensory nerve fibers that innervate the cranial vasculature contain calcitonin gene-related peptide (CGRP), substance P and neurokinin [8]. Endocannabinoid deficiency has been hypothesized to underlie the pathophysiology of migraine and several clinical studies [9] support this idea although biochemical studies providing a scientific basis for the potential efficacy of (endo)cannabinoids in migraine are really limited. In a previous study, it was reported that anandamide (AEA), an endogenous ligand to the CB receptor, inhibits CGRP-induced and nitric oxide (NO)-induced neurogenic dural vasodilatation, suggesting that AEA may be tonically released to modulate the trigeminovascular system [10]. Theoretically, the reduction of AEA levels, and thus the reduced inhibitory effect of endocannabinoid system (ECS), may contribute to facilitate/maintain central sensitization in chronic head pain, therefore providing an additional mechanism which contributes to CGRP release and NO production [11, 12]. Systemic administration of nitroglycerin (NTG), a NO donor, provokes spontaneous-like migraine attacks in migraine sufferers. NTG also induces a condition of hyperalgesia in the rat, through the activation of spinal and brainstem structures involved in nociception [13–15]; As such, NTG has been extensively used to investigate the neurobiological correlates of migraine pain, in rodents [13–15]; Recently, we have shown that NTG-induced hyperalgesia is associated with an alteration of ECS in some areas of rat brain [16]. In the mesencephalon, an increased activity of both the hydrolases that are involved in degradation of the 2-arachidonoylglycerol (2-AG) and AEA, fatty acid amide hydrolase (FAAH) and monoacylglycerol lipase (MAGL), has been observed, together with an increased density of CB binding sites in the mesencephalon. In the hypothalamus, NTG caused an increase in the activity of FAAH associated with an increase in density of CB binding sites, while, in the medulla only the activity of FAAH was increased [16]. In the present study we have investigated the possible role for AEA in the mechanisms mediating NTG-induced hyperalgesia in the formalin test, a well-established model of persistent somatic pain widely used in rats [17]. Additionally, we evaluated the effect of AEA on the cerebral expression of Fos protein elicited by NTG-induced hyperalgesia.

## Materials and methods

Adult male Sprague–Dawley rats, weighing 180–220 g, were used in the present investigation. Rats were randomly divided in groups formed by 4–6 animals each, and underwent the following experimental protocols.

### Drugs

AEA (Sigma), suspended in 4% Tween 80, was injected i.p. at a dose of 20 mg/kg, 30 min before the execution of the formalin test (see below). This dose was chosen based on the paper published by Jaggar et al. [18], where it was demonstrated that AEA (dose range 5–25 mg/kg) reduces the nociceptive behavior in the second phase of the formalin test. The 30-min interval, between AEA administration and the formalin test, was chosen on the basis of preliminary experiments showing a maximum antinociceptive effect for AEA at that time (data not shown).

AEA reduced ambulatory and non-ambulatory activities (rearing and grooming) and body temperature was significantly decreased by the dose utilized, as reported in a previous study [19].

Nitroglycerin (Astra Company, Italy), dissolved in saline alcohol and propylene glycol, was injected i.p. at a dose of 10 mg/kg.

### Behavioral response to formalin test

The formalin test is a well-established rat model of persistent somatic pain. Following injection of 100 μl of 1% formalin into the plantar surface of the right hind paw, the animals were placed, one at a time, in a plexiglas observation chamber (10 × 20 × 24 cm) in which a mirror (angled at 45°) allowed unimpeded observation of the animal’s paws. The total number of flinches/shakes per min was counted during the period from 1 to 5 min after injection (phase 1) and, subsequently, for 1-min periods at 5-min intervals during the period from 15 to 60 min (phase 2) after formalin injection. Flinches/shakes, characterized as a rapid and brief withdrawal or flexion of the injected paw, were readily identified. Incomplete formalin injection constituted an exclusion criterion for the study. The analgesic effect of AEA was evaluated by comparing the response to the formalin test of AEA-treated versus untreated rats, in basal conditions and following NTG administration (4 h after the administration).

#### Experimental groups

- Control (for NTG4 h): i.p. injection of saline 4 h before the formalin test;
- NTG4 h: i.p. injection of NTG 4 h before the formalin test;
- AEA + NTG4 h: i.p. injection of NTG (4 h before the formalin test) and administration of AEA 30 min before the formalin test;
- AEA: i.p. injection of AEA 30 min before the formalin test;
- Control (for AEA): i.p. injection of 4% Tween 80 (vehicle) 30 min before the formalin test.

### Fos immunohistochemistry

Animals were anaesthetized and perfused transcardially with saline and ice-cold 4% paraformaldehyde 4 h after NTG/saline administration. Brains were removed, post-fixed for 12 h in the same fixative and subsequently transferred in solutions of sucrose at increasing concentrations (up to 30%) during the following 72 h. Brains were cut at 50 μm on a freezing sliding microtome. Fos expression in the rat brain was detected by means of the immunohistochemical technique with a rabbit polyclonal antiserum directed against Fos protein (residues 4–17 of human Fos). Tissue sections were incubated for 48 h at 4°C with the Fos antibody (Oncogene). After thorough rinsing in buffer, sections were processed with the avidin biotin technique, using a commercial kit. Cells positively stained for Fos were visualized with nickel-intensified 3′,3′-diaminobenzidine tetra hydrochloride (DAB). After staining, sections were rinsed in buffer, mounted onto glass slides, air-dried and coverslipped.

#### Experimental groups

- NTG4 h: i.p. injection of saline 30 min before the NTG administration;
- Control (for NTG4 h): i.p. injection of saline 30 min before the saline administration;
- AEA + NTG4 h: i.p. injection of AEA, 30 min before the NTG administration;
- AEA: i.p. injection of AEA, 30 min before the saline administration;
- Control (for AEA): i.p. injection of 4% Tween 80 (vehicle) 30 min before the saline administration.

### Statistical evaluation

In the formalin test, the total number of flinches/shakes evoked by formalin injection was counted in phases I and II of the formalin test, as described above. Differences between groups were analyzed by Student’s *t* test and a probability level of <5% was regarded as significant.

For Fos expression, cell counts of individual nuclei were made from every sixth section throughout their rostrocaudal extent for each rat and its control. In order to avoid differences related to the asymmetrical sectioning of the brain, Fos-positive cells were counted bilaterally (3 sections for each nucleus) (Scion Image Analysis) and the mean value obtained from the two sides was used for the statistical analysis. Student’s *t* test for unpaired data was used to compare differences in the mean number of Fos-immunoreactive nuclei per cell group between controls and treatment groups. A probability level of <5% was regarded as significant.

## Results

### Anandamide and nitroglycerin-induced hyperalgesia at formalin test

**Fig. 1:**
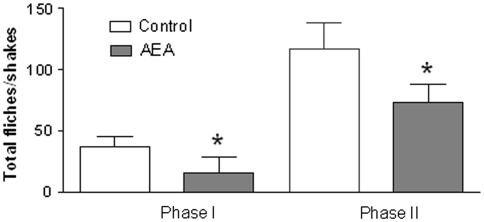
Effect of anandamide (AEA) treatment on hyperalgesia at the formalin test. Pre-treatment with AEA, 30 min before vehicle administration, significantly decreases the total number of flinches/shakes during phase I and II. *p < 0.05 versus control group. Data were expressed as mean ± SD

**Fig. 2:**
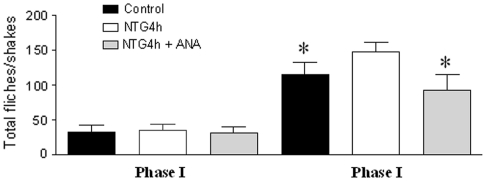
Effect of anandamide (AEA) pre-treatment on nitroglycerin-induced hyperalgesia at the formalin test. Pre-treatment with AEA, 30 min before nitroglycerin (NTG) administration, significantly decreases the total number of flinches/shakes during phase II. *p < 0.05 versus NTG4 h group. Data were expressed as mean ± SD

In the control group (for AEA), the injection of formalin resulted in a highly reliable, typical, biphasic pattern of flinches/shakes of the injected paw, being characterized by an initial acute phase of nociception within the first 5 min, followed by a prolonged tonic response from 15 to 60 min after formalin injection. AEA administration significantly reduced the nociceptive behavior in both phases of the formalin test (Fig. 1). NTG administration significantly increased the total number of flinches/shakes in phase II of formalin test, confirming previous reports [14, 15]. AEA pre-treatment significantly inhibited the nociceptive behavior induced by NTG administration during phase II of the test (Fig. 2).

### Anandamide and Fos expression in the animal model of migraine

**Fig. 3:**
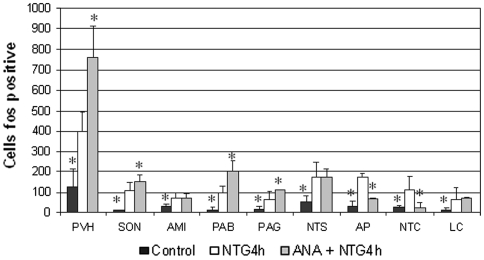
Pretreatment with anandamide (AEA) induced a significant increase of nitroglycerin-induced neuronal activation in several brain nuclei, which include paraventricular (PVH) and supraoptic nuclei (SON) of the hypothalamus, parabrachial nucleus (PAB), periaqueductal grey (PAG). By contrast, AEA induced a significant decrease of Fos expression in the nucleus trigeminalis caudalis (NTC) and area postrema (AP). *p < 0.05 versus NTG4 h group. Data were expressed as mean ± SD

**Fig. 4:**
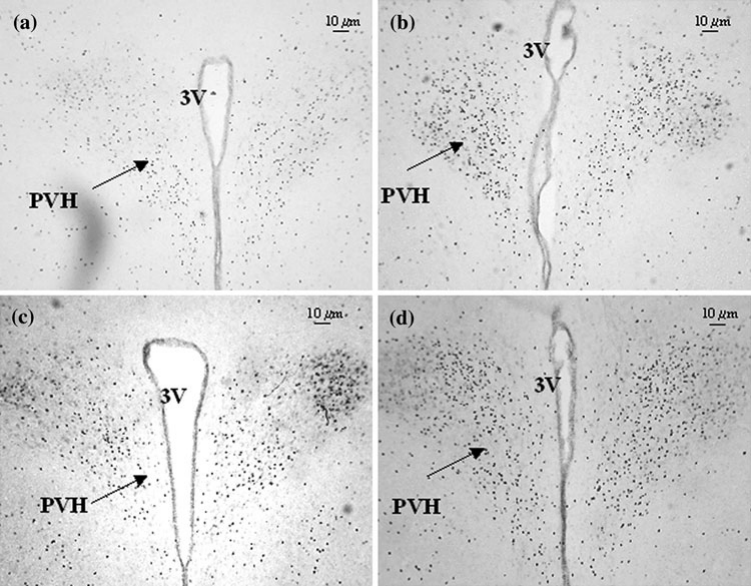
Micrographs of representative sections of the paraventricular nucleus of hypothalamus (PVH) of rats treated with nitroglycerin (**a**) or pre-treated with anandamide (AEA) before receiving nitroglycerin (**b**), or treated with saline (**c**) or AEA (**d**)

**Fig. 5:**
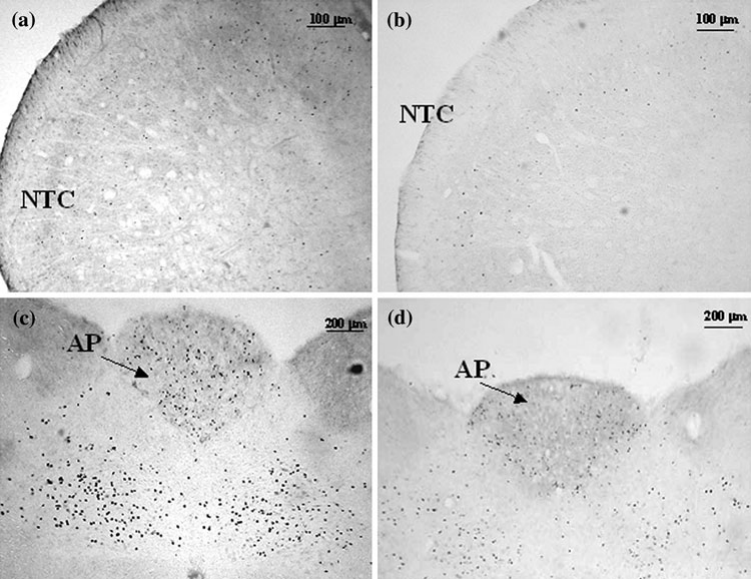
Micrographs of representative sections of the nucleus trigeminalis caudalis and area postrema of rats treated with nitroglycerin (NTG) (**a**–**c**) or pre-treated with anandamide (AEA) before receiving NTG (**b**–**d**). *AP* area postrema, *NTC* nucleus trigeminalis caudalis

**Fig. 6:**
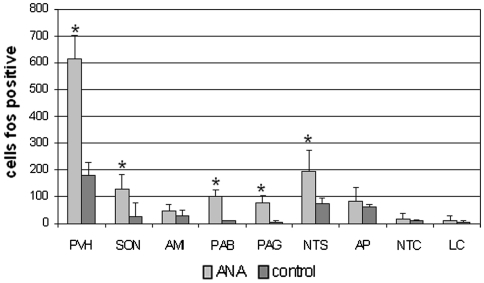
Anandamide administration induces a significant increase of Fos expression in several brain nuclei, which include paraventricular (PVH) and supraoptic nuclei (SON) of the hypothalamus, parabrachial nucleus (PAB) and periaqueductal grey (PAG). *p < 0.05 versus control group. Data were expressed as mean ± SD

In agreement with our previous findings [20, 21, 22], NTG administration induced neuronal activation in paraventricular (PVH) and supraoptic nuclei (SON) of the hypothalamus, central nucleus of the amygdala (CEA), ventrolateral column of the periaqueductal grey (PAG), parabrachial nucleus (PBN), locus coeruleus (LC), nucleus tractus solitarius (NTS), area postrema (AP) and nucleus trigeminalis caudalis (NTC). Pre-treatment with AEA significantly reduced the NTG-induced neuronal activation in NTC, confirming the results obtained in our previous study [16], and in AP, while the same treatment induced an increase of Fos expression in PVH, SON, PAG, PBN (Figs. 3, 4, 5). It is noteworthy that AEA administration *per se* increased significantly Fos expression in PVH, SON, PAG and PBN (Fig. 6), confirming previous studies. Indeed, when AEA and AEA + NTG groups were compared no differences were seen with regard to Fos expression in these latter nuclei.

## Discussion

### Anandamide and nitroglycerin-induced hyperalgesia

NTG activates specific cerebral nuclei and induces hyperalgesia through the intervention of selected neurotransmitters and neuromediators, with a specific time-pattern in rats [14, 15]. Endocannabinoid receptors have been identified in many of the NTG-activated areas [23, 24]. AEA induced a significant decrease in the nociceptive behavior during both phases of the formalin test in the animals treated with vehicle; furthermore, AEA abolished NTG-induced hyperalgesia in phase II of formalin test. These results are in accordance with previous observations showing that AEA, in the range of 10–100 mg/kg, has analgesic effects in hyper-acute somatic nociceptive models, such as the tail-flick and hot-plate tests [25, 26].

The mechanisms and targets underling the AEA-mediated modulation of NTG-induced hyperalgesia are not clearly understood. The anti-hyperalgesic effect of AEA observed in the formalin test could be localized at the spinal and supraspinal level. It was demonstrated that the antinociceptive effects of ∆^9^-tetrahydrocannabinol (∆^9^-THC) in the tail-flick test are attenuated following spinal transection, showing that also supraspinal sites may play an important role in CB antinociceptive action [27]. Another study showed that microinjection of CB agonists into the dorsolateral PAG produce antinociception [28]. CB1 receptors are localized on fibers in the spinal trigeminal tract and in the NTC [6]. Therefore, it is also possible that AEA exerts a direct effect upon trigeminal neurons [10] to cause inhibition of CGRP release from central terminals of primary afferent fibers, and to reduce the nociceptive behavior. NTG induces CGRP release from NTC for a period of 4 h after its administration, while formalin injection induces an ipsilateral decrease in CGRP in the NTC 1 h later [29, 30]. AEA interacts mainly with CB1 receptors [31], although CB2 receptors located in the lower brainstem may also be involved [32, 33]. In line with this idea, it was shown that selective activation of CB2 receptors suppresses spinal Fos protein expression and pain behavior in a rat model of inflammation [5].

### Anandamide and Fos expression in the animal model of migraine

The present investigation shows AEA-induced Fos immunoreactivity in a wide variety of cerebral nuclei, whose distribution is similar to previous studies by other groups [34, 35]. Pre-treatment with AEA selectively inhibited NTG-induced Fos expression, in the NTC and AP, areas involved in the generation and modulation of migraine pain. No definite conclusion can be drawn with regard to the effect of AEA on the other nuclei that are known to be activated by NTG (PVH, SON, PAG, and PBN) because AEA *per se* induces an intense Fos expression in these structures that outweighs NTG-induced Fos expression.

The finding regarding the inhibition of NTG-induced Fos expression in the NTC and AP seems particularly relevant for the role of AEA in migraine. With regard to NTC, activation of CB receptors may influence trigeminovascular neuronal firing by reducing expression of Fos protein, as suggested by our previous study [16]. Indeed, CB1 receptors are expressed also on axon terminals of primary sensory neurons, i.e. in the nociceptive areas of spinal cord, DRG and trigeminal ganglia, and their expression is partially co-localized with CGRP and substance P [3]. AEA is capable of inhibiting capsaicin-evoked CGRP release from terminals of primary afferent fibers of spinal cord to modulate neurotransmitters release [36]. Our results are in agreement with data obtained in another animal model of migraine, where it was shown that activation of CB1 receptor reduces Fos immunoreactivity induced after activation of the ophthalmic division of the trigeminal nerve, in neurons of the NTC [37]. Additionally, AEA might inhibit neuronal activation in the NTC also via CB2 receptors [32]. Also the inhibitory effect of AEA in AP is relevant for migraine, when considering that nausea and vomiting are the most frequently accompanying symptoms of migraine pain. AP indeed is an important area for the control of autonomic functions. Our results are in agreement with data from Van Sickle et al. [33] that have reported a reduction of Fos expression induced by emetic stimuli in the AP following ∆^9^-THC administration. CB1 receptors play a more important role in the brainstem, as compared to VR1 receptors, in the control of emesis, indicating that endogenously released endocannabinoids/endovanilloids inhibit emesis preferentially via CB1 receptors [37].

## Conclusions

The anti-nociceptive effect of AEA on NTG-induced hyperalgesia is unequivocally demonstrated in this study, and it seems that the exact localization of this effect is the NTC. Furthermore, AEA inhibits the effect of NTG on AP, the emetic area par excellence.

By combining our data with the findings available from the literature, we can hypothesize that a dysfunction of the endocannabinoid system may contribute to the development of migraine attacks and that a specific pharmacological modulation of CB1 and CB2 receptors may be useful for the treatment of migraine pain, without deleterious effects, as well as of specific associated symptoms (nausea, *in primis*).
